# Elevated Negative Affectivity and Psychoticism in individuals with active Nonsuicidal Self-Injury (NSSI) compared with individuals with previous or no NSSI

**DOI:** 10.1186/s40479-026-00373-z

**Published:** 2026-09-22

**Authors:** Christina Thylander, Magnus Nilsson, Danielle van Westen, Johannes Björkstrand, Sofie Westling

**Affiliations:** 1 Psychiatry, Department of Clinical Sciences Malmö, Lund University, Lund, Sweden; 2 Department of Psychiatry, Skåne University Hospital, Lund, Sweden; 3 Image and Function, Skane University Hopsital, Lund, Sweden; 4 Diagnostic Radiology, Institution for Clinical Sciences Lund, Lund University, Lund, Sweden; 5 Department of Psychology Lund, Lund University, Lund, Sweden

**Keywords:** Nonsuicidal Self-Injury (NSSI), Diagnostic and Statistical Manual of Mental Disorders, Fifth Edition (DSM-5), Personality Inventory for DSM-5 (PID-5)

## Abstract

**Background:**

Non-suicidal self-injury (NSSI) is a transdiagnostic symptom associated with substantial clinical burden and increased risk of suicidal behavior. Although NSSI occurs across a wide range of psychiatric disorders, research has consistently linked it to maladaptive personality traits. However, most studies have compared individuals with NSSI to healthy controls, limiting our understanding of which personality traits are specifically associated with NSSI. The inclusion of clinical control groups may therefore help clarify which personality traits are associated with NSSI rather than with psychopathology more broadly. This study aimed to investigate whether individuals with psychiatric disorders and active NSSI differ in personality traits, as assessed with the Personality Inventory for DSM-5 (PID-5), compared to those with prior but no active NSSI and those without a history of NSSI.

**Methods:**

231 individuals at a psychiatric outpatient unit completed the PID-5 and the Inventory of Statements About Self-injury and were assessed with regards to demographic information, psychiatric illness and previous suicide attempts. Symptom levels of depression and anxiety were measured. Participants were divided into groups of active NSSI (≥ 5 episodes in the past 12 months; *n* = 123), previous NSSI (≥ 5 episodes, but none in the past 12 months; *n* = 68) and no previous history of NSSI (*n* = 40).

**Results:**

Participants with active NSSI scored significantly higher in negative affectivity (F(2, 224) = 10.33, *p* < .001, η_p_² = 0.08) and psychoticism (F(2, 224) = 8.91, *p* < .001, ηp² = 0.07), compared to those with a previous history of NSSI and those with no previous history of NSSI. The analyses were adjusted for age, sex assigned at birth and symptoms of depression and anxiety.

**Conclusions:**

Active NSSI seems to be associated with increased traits of negative affectivity and psychoticism. This study contributes with new knowledge regarding NSSI and personality traits, which may guide future research on mechanisms, etiology, and treatment approaches.

**Trial registration:**

The study was registered on Clinical Trials: NCT04905797, registration date April 29, 2021.

**Supplementary Information:**

The online version contains supplementary material available at https://doi.org/10.1186/s40479-026-00373-z.

## Introduction

Nonsuicidal Self-Injury (NSSI) - defined as the intentional and direct damage to one’s own bodily tissue without lethal intent and for reasons not socially sanctioned [1, 2] - is common, not only within healthcare settings but also among individuals with no other indications of mental illness [3–6]. Lifetime prevalence studies estimate that 3–6% of adults engage in NSSI [5, 7, 8] with significantly higher rates observed in clinical populations [6, 9, 10]. NSSI is recognized as a transdiagnostic symptom that occurs across a broad spectrum of psychiatric disorders. In clinical populations NSSI is, for example, prevalent in individuals with personality disorders and is strongly associated with mood disorders such as anxiety and depression [10, 11]. Furthermore, NSSI is recognized as one of the strongest predictors of future suicide [12] and is associated with premature mortality, both due to suicide and other health-related conditions [13].

NSSI is commonly associated with Borderline Personality Disorder (BPD), as it is the only psychiatric diagnosis that includes NSSI as a diagnostic criterion according to DSM-5 (the *Diagnostic and Statistical Manual of Mental Disorders*,* Fifth Edition*) [14]. However, the transdiagnostic nature of NSSI suggests that it may be linked not only to specific psychiatric disorders but also to broader personality characteristics that cuts across diagnostic categories. Consequently, dimensional models of personality pathology may offer a useful framework for investigating personality correlates of NSSI. In 2013 the American psychiatric Association launched the DSM-5, where a dimensional model for personality disorders was introduced, the Alternative Model for Personality Disorders (AMPD) [14]. AMPD conceptualizes maladaptive personality traits across five domains: Negative affectivity, Detachment, Antagonism, Disinhibition and Psychotisism, measured by the Personality Inventory for DSM-5 (PID-5).

The AMPD was developed in alignment with the Five-Factor Model (FFM), which is bult upon a lexical model, measuring normal personality traits, also across five domains: neuroticism, agreeableness, extraversion, openness to experience, and conscientiousness [15]. Although the Five-Factor Model (FFM) primarily describes normative personality traits and the Alternative DSM-5 Model for Personality Disorders (AMPD) focuses on maladaptive personality characteristics, growing evidence indicates considerable convergence between the two models [16]. Four trait-domain pairs have been identified, with Negative Affectivity corresponding to Neuroticism, whereas Detachment, Disinhibition, and Antagonism broadly reflect maladaptive variants of low Extraversion, low Conscientiousness, and low Agreeableness, respectively [16].

NSSI has been studied from the perspective of both models. Individuals with a history of NSSI have been found to report higher levels of negative affectivity and neuroticism in several studies. A study of 100 Italian adolescent inpatients with NSSI revealed a significant but modest association between negative affectivity and clinical ratings of NSSI [17]. This study focused on hospitalized adolescents with moderate to severe NSSI, limiting the generalizability of its findings. Brown [18] included 238 college students and found that the 59 individuals engaging in NSSI tended to score high in neuroticism. Similarly, MacLaren et al. [19] examined 153 Canadian psychology students and reported that individuals with NSSI demonstrated higher scores on neuroticism, compared to those with no NSSI. These findings align with an Italian study of 270 young adults attending youth mental services, which found that those with NSSI and/or suicidal ideation had significantly higher scores in negative affectivity [20]. Low agreeableness and low conscientiousness have also emerged as common traits among individuals with NSSI in non-clinical samples. MacLaren et al. [19] noted that individuals with frequent NSSI scored lower in both agreeableness and conscientiousness, and in addition Brown [18] reported similar patterns. Findings regarding openness to experience have been more variable. Goldstein et al. [21] observed that individuals with deliberate self-harm (i.e. self-injurious behavior with and without suicidal intent) scored higher on openness (in a sample of 320 university students), while MacLaren et al. [19] found that only those with low-frequency NSSI exhibited elevated openness, as compared to those with frequent NSSI.

Most prior research has compared individuals with and without NSSI, without distinguishing between current NSSI and past. This distinction is clinically meaningful. In psychiatric populations, NSSI may occur in a substantial proportion of individuals at some point during the course of illness [6]. While many psychiatric patients may engage in NSSI during some period of elevated distress, only some individuals continue to engage in NSSI over time. Consequently, lifetime history of NSSI may capture vulnerability to engage in the behavior, whereas current NSSI may reflect factors involved in its maintenance and persistence. Individuals with past NSSI represent a particularly informative group, as they share a history of the behavior but have subsequently discontinued it. Examining differences between current and past NSSI may therefore help identify personality traits associated with the persistence of NSSI beyond general vulnerability to self-injury. A clinical group with no history of NSSI helps distinguish NSSI-specific traits from characteristics associated with general psychopathology. Such knowledge may improve our understanding of trait-related factors associated with the maintenance of NSSI and inform more targeted interventions aimed at preventing chronic or recurrent self-injurious behavior.

Research specifically examining traits associated with the persistence, rather than merely the occurrence, of NSSI remains limited. Nevertheless, prospective studies indicate that several dispositional characteristics may be relevant. Borderline personality features, together with greater initial NSSI severity, have been identified as predictors of continued NSSI engagement [22]. Different facets of impulsivity may also be relevant at different stages of the behavior: negative urgency predicted NSSI onset, whereas lack of perseverance predicted the maintenance of NSSI status after controlling for prior NSSI behavior [23]. Lower perceived emotion-regulation capability has also been found to differentiate persistent from ceased NSSI in emerging adulthood [24]. Collectively, these findings point to dispositional vulnerabilities involving negative emotionality and impaired self-regulation. However, prior research has rarely examined whether the broader maladaptive trait domains of the AMPD distinguish current from past NSSI, particularly within psychiatric populations.

The AMPD provides a useful framework for this question because its maladaptive trait domains map onto theoretically relevant mechanisms of NSSI. Negative affectivity reflects emotional instability and proneness to intense negative emotions, which is consistent with models describing NSSI as a strategy for regulating aversive affect. In the four-function model, NSSI may be maintained by automatic negative reinforcement, for example through reduction or escape from aversive emotional states [25]. Daily-life studies further support the central role of negative affect in NSSI, although affective relief after NSSI is not always sustained [26]. Disinhibition reflects impulsivity, risk-taking, poor planning, and difficulties inhibiting behavior under emotional distress. It is relevant since NSSI may function as a rapidly available strategy for reducing negative affect, while poor inhibitory control may increase the likelihood that NSSI urges are acted upon. Consistent with this, impulsivity has repeatedly been suggested as important in NSSI, although findings vary depending on how impulsivity is measured [27, 28].

Antagonism reflects hostility, low empathy, mistrust, and interpersonal conflict, which may be relevant because NSSI often occurs in interpersonal contexts [29]. Furthermore, antagonism may be relevant because such traits overlap with low agreeableness, a personality dimension that has been shown to influence the development of the working alliance in patients with borderline personality disorder [30]. Taken together, previous studies suggest that NSSI is associated with maladaptive personality traits, particularly negative affectivity/neuroticism, disinhibition/conscientiousness, and, to a lesser extent, antagonism/low agreeableness.

When personality traits are assessed using a self-report measure, current symptoms of depression and anxiety may influence individuals’ self-perception and, consequently, how they evaluate and report their own personality characteristics. This is particularly relevant in psychiatric populations, where depressive and anxiety disorders are highly prevalent. Accordingly, the potential influence of current affective symptoms should be considered when interpreting self-reported personality traits. Furthermore, depression and anxiety share conceptual overlap with several maladaptive personality traits, particularly those related to negative affectivity. Consequently, observed associations between PID-5 domains and NSSI could potentially reflect current symptom burden rather than more enduring personality characteristics.

The aim of the present study was to investigate, in a clinical population, whether individuals with current NSSI differ in maladaptive personality traits, measured with the PID-5, from individuals with past NSSI and those without a history of NSSI. We preregistered the hypothesis that individuals with current NSSI would score higher on negative affectivity than the other two groups. Group differences in antagonism, disinhibition, detachment, and psychoticism were examined exploratively, as were differences between individuals with past NSSI and those without a history of NSSI.

## Methods

### Design and sample

The study design was cross-sectional. Apart from the hypothesis that individuals with current NSSI would score higher on negative affectivity than the other two groups, the preregistration also included directional hypotheses for antagonism and disinhibition. However, the preregistered direction of these hypotheses resulted from a misinterpretation of the respective scales. This error was identified after preregistration and was not corrected through a preregistered amendment prior to data inspection and analysis. Analyses of antagonism and disinhibition are therefore treated as exploratory in the present study.

Participants were individuals **≥** 18 years old, recruited from two general psychiatric outpatient care units in Lund, a university town of approximately 130.000 inhabitants in southern Sweden. A list of all registered patients as of 12 April 2021, including their main diagnoses was distributed by the administrative unit. Patients with the registered main diagnosis psychotic disorder or intellectual disability were directly excluded from the sample. Potential participants were contacted in ascending order of age, starting at 18 years, until the required sample size according was reached. This strategy is based primarily on the fact that NSSI is more common among younger adults than older adults [31]. Participants were initially contacted by phone/email/text and provided with brief information about the study. Those expressing interest in participating received detailed written information via email, and an appointment was scheduled. At the appointment, participants were given comprehensive oral and written information, and any remaining questions were addressed. A total of 240 participants were recruited. Each participant received 400 Swedish kronor (≈35 €) in cash as compensation.

Participants were enrolled between April 30, 2021, and August 26, 2022.

The data collection was conducted by the first author with the assistance of three medical students.

### Data collection

Participants used a laptop to fill out demographic data and databased forms. Participants reported their sex assigned at birth (female, male, or intersex) and their gender identity (woman, man, non-binary, agender, genderqueer, transgender, other, or prefer not to disclose). All forms were completed the same day, including as follows:

1. *The self-administered form Personality Inventory for DSM-5 (PID-5)* [32, 33] is a questionnaire, developed as a part of the AMPD to assess madadaptive personality traits. It concists of 220 items rated on a 4-point Likert scale ranging from 0 to 3, labeled “*Very false or Often False*”, “*Sometimes or Somewhat False*”, “*Sometimes or Somewhat True*” and “*Very True or Often True*”. Answers of each item are scored according to a calculated form, resulting in scores for 25 personality trait facets that can be summed up in five personality trait domains (negative affectivity, detachment, antagonism, disinhibition and psychoticism), see Table 1.
  The Personality Inventory for DSM-5 (PID-5) demonstrated good to excellent internal consistency in the present sample. Cronbach’s alpha coefficients were 0.93 for Negative Affectivity, 0.94 for Psychoticism, 0.93 for Detachment, 0.89 for Antagonism, and 0.91 for Disinhibition.

**Table 1:** *Definitions of personality trait domains as stated in DSM-5* [14]

Domain	Definition
Negative Affectivity	Frequent and intense experiences of high levels of a wide range of negative emotions (e.g., anxiety, depression, guilt/shame, worry, anger) and their behavioral (e.g., self-harm) and interpersonal (e.g., dependency) manifestations.
Detachment	Avoidance of socioemotional experience, including both withdrawal from interpersonal interactions (ranging from casual, daily interactions to friendships to intimate relationships) as well as restricted affective experience and expression, particularly limited hedonic capacity.
Antagonism	Behaviors that put the individual at odds with other people, including an exaggerated sense of self-importance and a concomitant expectation of special treatment, as well as a callous antipathy toward others, encompassing both an unawareness of others’ needs and feelings and a readiness to use others in the service of self-enhancement.
Disinhibition	Orientation toward immediate gratification, leading to impulsive behavior driven by current thoughts, feelings, and external stimuli, without regard for past learning or consideration of future consequences.
Psychoticism	Exhibiting a wide range of culturally incongruent odd, eccentric, or unusual behaviors and cognitions, including both process (e.g., perception, dissociation) and content (e.g., beliefs).

2. *The Inventory of Statements About Self-injury (ISAS)* [34, 35] is a questionnaire for measure of frequency and functions of NSSI primarily causing damage to the body tissue, completed with the possibility to fill out information about any kind of NSSI but without suicidal intention. The form is divided into two sections, the first concerning life-time prevalence of 12 different kinds of NSSI and the completing item labelled “other”, the second part exploring functions of NSSI. For this study, only the first section was used. As a complement for this study, we added questions about how many of the NSSI occasions that occurred within the last 3 months and within the last year.
3. *The Generalized Anxiety Disorder 7-item scale (GAD-7)* [36] is a self-administered questionnaire consisting of seven items designed to screen for Generalized Anxiety Disorder (GAD) and measure anxiety levels over the past 14 days. Each item begins with the prompt: *“Over the last two weeks*,* how often have you been bothered by the following problems?”* and is rated on a 4-point Likert scale. The response options range from 0 (*Not at all*), 1 (*Several days*), 2 (*More than half the days*), to 3 (*Nearly every day*).
  In the present sample, the GAD-7 demonstrated good internal consistency (Cronbach’s alpha = 0.88).
4. *The Montgomery-Åsberg Depression Rating Scale – Self-administered (MADRS-S)* [37] is a 9-item self-report instrument designed to evaluate symptom severity in major depressive disorder. It assesses nine dimensions of depression experienced over the past three days: mood, anxiety, sleep, appetite, concentration, initiative, emotional engagement, pessimism, and will to live. Each item is scored on a 7-point ordinal scale ranging from 0 (no symptoms) to 6 (severe symptoms). In the present sample, the MADRS-S demonstrated good internal consistency (Cronbach’s alpha = 0.86).

Clinical routine at the study site is to establish psychiatric diagnoses through a comprehensive clinical assessment integrating structured interview findings (e.g. from MINI 7.0), psychiatric history, current symptom presentation, level of functioning, and longitudinal clinical information. Information regarding current diagnoses (to complete the original list only containing main diagnoses) previous suicide attempts and medications were extracted from medical records by the first author to describe the cohort. Any participant who had ever been diagnosed with psychotic disorders or intellectual disability was excluded, regardless of whether that diagnosis was recorded as primary or secondary diagnosis. For one participant, there were indications of insufficient diagnostic evaluation in the medical records. Consequently, a MINI 7.0 (Mini International Neuropsychiatric Interview 7.0) [38] was administered by a senior resident in psychiatry (author CT).

The choice to register all current diagnoses, and not only the main diagnosis, is based on the fact that in the clinic main diagnosis tends to shift between doctors’ appointments and over time, and is not considered representative of the patients’ symptom profile.

Study data were collected and managed using Research Electronic Data Capture (REDCap) [39, 40], an electronic data capture tool hosted at Lund University. REDCap is a secure, web-based software platform designed to support data capture for research studies.

### Group classification

Participants were categorized into three groups based on whether they reported NSSI or not, and if applicable the time elapsed since their last occurrence of NSSI:

- *Active NSSI*: Individuals with psychiatric disorders who engaged in five or more NSSI episodes within the past 12 months (*n* = 123), based on the criteria for NSSI Disorder as outlined in DSM-5, Section III [14].
- *Previous NSSI*: Individuals with psychiatric disorders who had a history of NSSI, based on the criteria for NSSI Disorder as outlined in DSM-5, Section III [14], but not in the past 12 months (*n* = 68).
- *No NSSI history*: Individuals with psychiatric disorders and no lifetime history of NSSI or a maximum of four lifetime episodes (*n* = 40).

### Missing data and exclusions

**Fig. 1:**
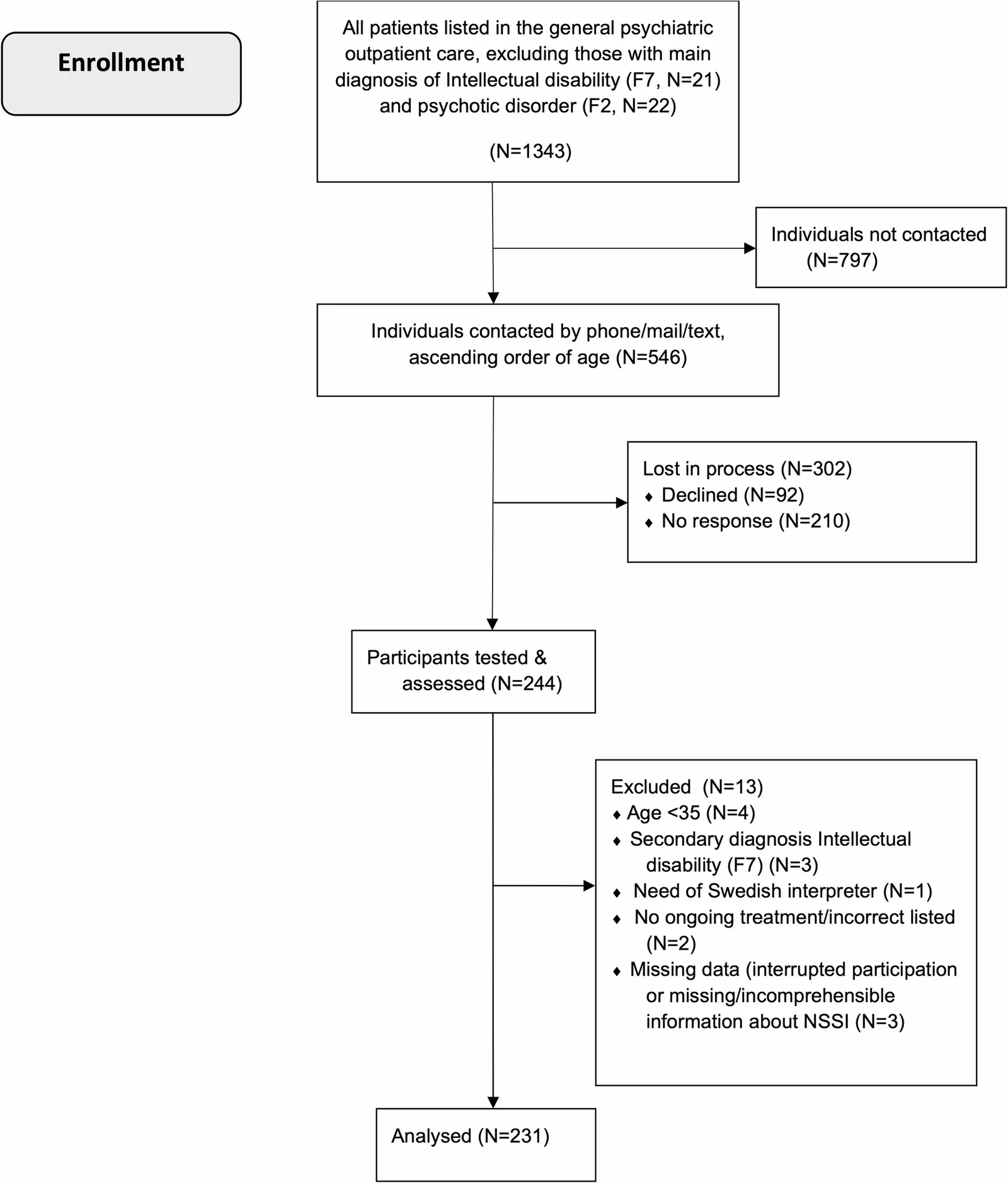
STROBE flowchart showing enrollment and exclusion criteria

Three participants were after inclusion found to have a secondary diagnosis of intellectual disability and were therefore excluded. One participant was unable to understand and use the Swedish language without significant difficulties, and two participants had no ongoing treatment at the general outpatient care unit and were therefore also excluded. Three participants were excluded due to missing data (Fig. 1). Finally, 231 participants were included in the final analyses. Thirty participants in total had left at most two questions unanswered in PID-5, GAD7 and MADRS-S. The inter-item consistency and reliability (Cronbach’s alpha) are high in all three questionnaires [41–43]. Since only single answers were missing, mean values were imputed.

### Data analysis plan

Descriptive statistics were calculated for all study variables. Continuous variables are presented as means and standard deviations, whereas categorical variables are presented as frequencies and percentages. Group differences in demographic and clinical characteristics were examined using one-way analyses of variance (ANOVA) for continuous variables and chi-square tests for categorical variables.

To examine differences in personality trait domains across NSSI groups, five separate analyses of covariance (ANCOVAs) were conducted, with NSSI status (active NSSI, previous NSSI, no lifetime history of NSSI) entered as the fixed factor and each PID-5 domain (Negative Affectivity, Detachment, Antagonism, Disinhibition, and Psychoticism) serving as the dependent variable. PID-5 domain scores were standardized (z-scores) prior to analysis to facilitate comparisons across domains. Age, sex assigned at birth, anxiety symptom severity (GAD-7), and depressive symptom severity (MADRS-S) were included as covariates. Adjusted means, standard errors, and 95% confidence intervals are reported.

Prior to analysis, ANCOVA assumptions were evaluated. Normality of residuals was assessed using Shapiro–Wilk tests and visual inspection of histograms and Q–Q plots. Homogeneity of variance was examined using Levene’s tests. Homogeneity of regression slopes was assessed by testing interactions between NSSI group and each covariate (age, sex assigned at birth, anxiety symptoms, and depressive symptoms). Influential observations were evaluated using standardized residuals and Cook’s distance.

Significant omnibus effects were followed by Bonferroni-adjusted pairwise comparisons. Effect sizes are reported as partial eta-squared (η²p). To account for multiple testing across the five ANCOVAs, a Bonferroni-corrected significance threshold of *p* < .01 was applied. All tests were two-tailed. Analyses were conducted using IBM SPSS Statistics version 30 [44].

### Ethical considerations

Before inclusion, oral and written information about the purpose of the study was provided and each participant gave their written informed consent. The study was approved by the Swedish Ethical Review Authority (2020–02732).

### Use of AI-assisted language editing

ChatGPT-5.3 Instant (OpenAI) was used solely for language editing and improvement of readability. No AI tools were used for data analysis, interpretation, or generation of scientific content. The authors reviewed, edited and refined the text and take full responsibility for the content.

## Results

### Demographic and clinical characteristic data

Clinical and demographic characteristics of the cohort are presented in Table 2. Both the group of individuals with active NSSI and the group with previous NSSI were dominated by female sex (χ^2^ (2) = 19.58, *p*<.001), while the group of no NSSI were equally divided between male and female participants. Age ranged from 18 to 35 years and did not differ significantly between the groups (Kruskal-Wallis Test H (2) = 5.52, *p*=.063). The main part of participants from all groups were born in Sweden (90.48%), and no significant difference in country of birth was observed between the groups (χ² (2) = 2.84, *p* = .241). Levels of education did not differ significantly between the three groups, (χ^2^ (8) = 5.90, *p*= .658).

**Table 2:** Demographic characteristics, current diagnoses, suicide attempts, scores of depression and anxiety in the three groups

	Active NSSI *n* = 123		Previous NSSI *n* = 68	No NSSI *n* = 40
**Age (years; M, SD)**	23.69 (4.20)		24.93 (4.69)	25.13 (4.44)
Min-max (years)	18–34		18–35	19–35
**Sex assigned at birth** (n,%)				
Female	104 (84.55)^1^		49 (72.06)^1^	20 (50.00)
Male	19 (15.45)		19 (27.94)	20 (50.00)
**Gender identity** (n,%)				
Woman	94 (76.42)		46 (67.65)	20 (50.00)
Man	15 (12.20)		18 (26.47)	19 (47.50)
Non-binary	4 (3.25)		2 (2.94)	
Agender	2 (1.63)		1 (1.47)	
Transgender	6 (4.88)			1 (2.50)
Another identity not listed above	1 (0.81)			
More than one of the above listed*	2 (1.63)		1 (1.47)	
**Country of birth** (n,%)				
Sweden	110 (89.43)		60 (88.24)	39 (97.50)
Other	13 (10.57)		8 (11.76)	1 (2.50)
**Highest form of completed education** (n,%)				
Not completed high school	14 (11.38)		13 (19.12)	4 (10.00)
High school	47 (38.21)		26 (38.24)	18 (45.00)
Post-secondary education (not university/college)	6 (4.88)		4 (5.88)	1 (2.50)
University / college studies	37 (30.08)		18 (26.47)	14 (35.00)
University / college degree	19 (15.45)		7 (10.29)	3 (7.50)
**Current psychiatric diagnoses** (n,%)				
Anxiety and stress-related disorders	61 (49.59)		28 (41.18)	17 (42.50)
Attention-deficit/hyperactivity disorder (ADHD)	58 (47.15)		38 (55.88)	25 (62.50)
Mood disorders	50 (40.65)		37 (54.41)	13 (32.50)
Autism spectrum disorder	24 (19.51)		15 (22.06)	5 (12.50)
Personality disorders	23 (18.70)		15 (22.06)	2 (5.00)
Borderline Personality Disorder (BPD)	17 (13.82)		11 (16.18)	
Obsessive-Compulsive Personality Disorder (OCPD)	2 (1.63)			1 (2.50)
Avoidant Personality Disorder	1 (0.81)			
Dependent Personality Disorder			1 (1.47)	
Unspecified Personality Disorder	3 (2.44)		3 (4.41)	1 (2.50)
Post-traumatic stress disorder (PTSD)	15 (12.20)		3 (4.41)	4 (10.00)
Eating disorders	12 (9.76)		4 (5.88)	1 (2.50)
Gender dysphoria	7 (5.69)		1 (1.47)	1 (2.50)
Substance use disorders	5 (4.07)		2 (2.94)	1 (2.50)
Unspecified mental disorders and others	3 (2.44)		1 (1.47)	1 (2.50)
**Score MADRS-S** (M, SD) Min-max (score)	21.37 (8.60)^2^ 1–44		17.72 (8.75) 2–50	14.60 (9.07) 0–36
**Score GAD-7** (M, SD) Min-max (score)	10.41 (5.37)^3^ 0–21		8.38 (5.24) 0–21	6.75 (5.42) 0–21
**Suicide attempts** (M, SD) Min-max (number of attempts)	1.89 (0.54)^4^ 0–45		1.18 (0.26)^5^ 0–10	0.18 (0.08) 0–2

^1^Female sex > Male sex, ^2^Active NSSI > Previous NSSI & No NSSI, ^3^Active NSSI > Previous NSSI & No NSSI, ^4^Active NSSI > No NSSI, ^5^Previous NSSI > No NSSI

* One individual identified as genderqueer and transgender, one individual identified as non-binary and transgender, and one individual identified as woman and transgender

Current diagnoses were grouped as seen in Table 2. The most common diagnoses were anxiety and stress-related disorders, attention-deficit/hyperactivity disorder and mood disorders. The distribution of current psychiatric diagnoses did not differ significantly across the three groups (χ² (18) = 23.38, Monte Carlo *p* = .180).

Regarding differences in symptoms of depression, MADRS-S scores differed significantly between groups (F(2, 228) = 10.32, *p* < .001), the effect size was in the medium range according to Cohen’s guidelines (η² =0.08). Post hoc analyses using Tukey’s honest significant difference (HSD) showed that the group with active NSSI had significantly higher MADRS-S scores compared to both the group with previous NSSI (mean difference = 6.77, *p*<.001) and the no-NSSI group (mean difference = 3.65, *p*=.017). No significant difference was observed between the previous NSSI and no-NSSI groups. Results of differences in symptoms of anxiety were similar. GAD-7 scores differed significantly between the three groups (F(2, 228) = 8.14, *p*<.001), and the effect size was in the medium range (η² = 0.07). Post hoc analyses using Tukey’s HSD indicated that the active NSSI group reported significantly higher scores compared to both the previous NSSI group (mean difference = 3.66, *p* < .001) and the no-NSSI group (mean difference = 2.02, *p* = .034). No significant difference was observed between the previous and no NSSI groups.

The number of suicide attempts in the whole sample ranged from 0 to 45 (M = 1.39, SD = 4.54) and differed significantly between the three groups (Kruskal-Wallis H [2] = 11.96, *p* = .003). The group of active NSSI had the highest mean value and the widest range of attempts, with two extreme outliers of 42 and 45 attempts (see Table 2). Post hoc pairwise comparisons with Bonferroni correction was performed to explore group variance. This showed a significant difference of suicide attempts between the group of active NSSI and no NSSI (*p*=.004), and between the group of previous NSSI and no NSSI (*p*=.006). No significant differences between the group of active and previous NSSI was seen.

Seven participants were found to have attempted suicide 10 or more times, 5 of them in the group of active NSSI whereas the remaining 2 in the group of previous NSSI. 22 participants had a history of suicide attempts requiring somatic medical care − 14 of them in the group of active NSSI, 7 in the group of previous NSSI and 1 participant in the group of no NSSI.

### Analyses of personality domains and NSSI

Assumption checks generally supported the use of ANCOVA. Residuals were approximately normally distributed for the Negative Affectivity and Disinhibition models and showed only minor deviations from normality for the Psychoticism and Detachment models. More substantial departures from normality were observed for the Antagonism model. Levene’s tests supported homogeneity of variance for all models except Detachment, for which a modest violation was observed (*p* = .042). No significant interactions were found between NSSI group and any covariate (all ps > 0.05), indicating that the assumption of homogeneity of regression slopes was met. Four observations in the Antagonism model exhibited standardized residuals greater than + 3; however, Cook’s distance values were low across all models (maximum = 0.09), indicating that no individual observation exerted undue influence on model estimates. Sensitivity analyses excluding observations with standardized residuals greater than + 3 yielded the same pattern of results.

**Fig. 2:**
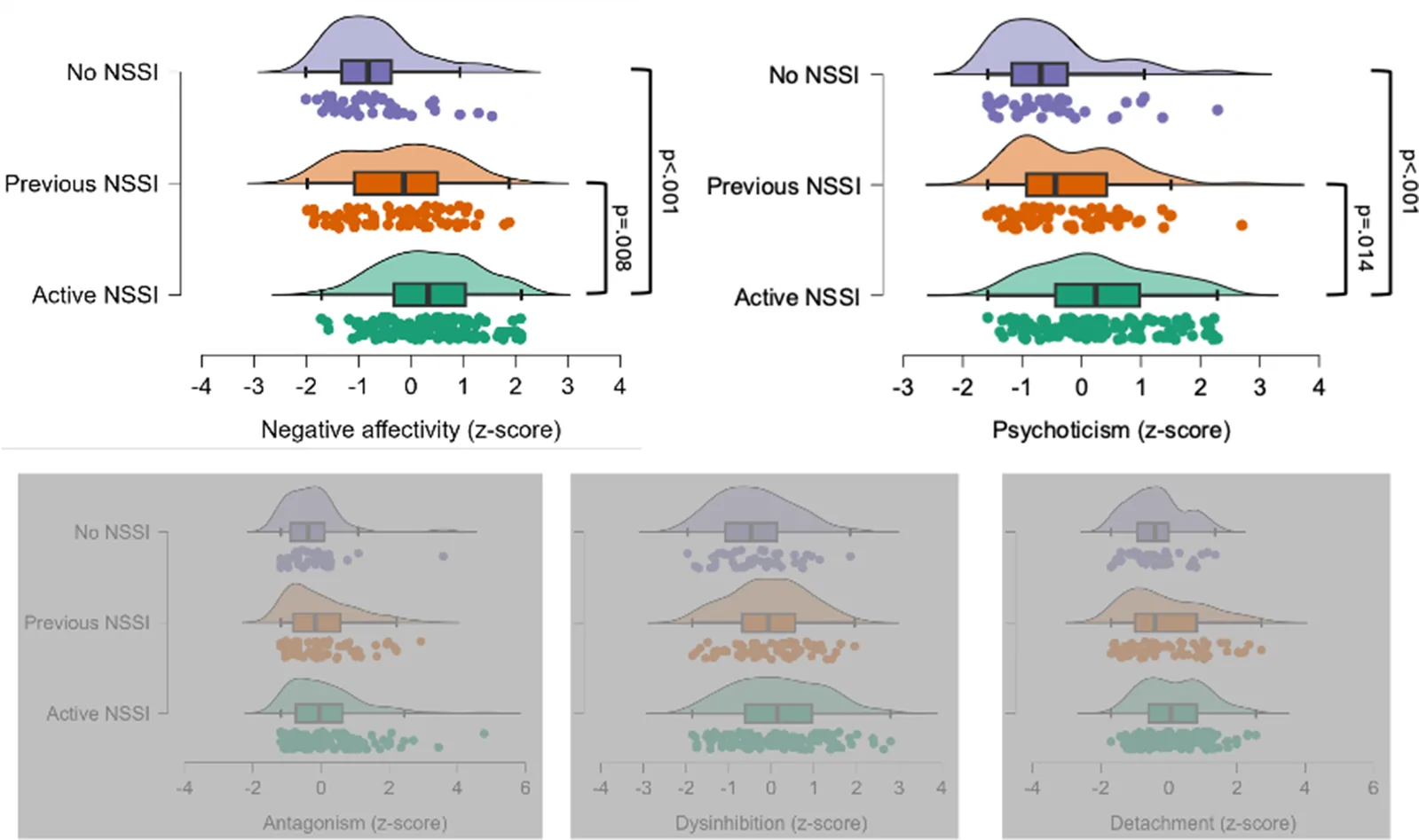
Analyses of personality domains and NSSI. Groups of active NSSI, previous NSSI and no NSSI analyzed with analysis of covariance (ANCOVA) for the five different personality traits. Displayed values reflect estimated marginal mean adjusted for covariates, evaluated at the following values: age = 24.30, anxiety (as measured with GAD-7) = 9.18, sex = 1.25 and depression (as measured with MADRS-S) = 19.12. Points and error bars display means and 95% confidence intervals. The group of active NSSI had significantly higher levels of negative affectivity (Bonferroni-adjusted pairwise comparisons for: active NSSI and no NSSI *p*<.001; active NSSI and previous NSSI *p*=.008) and psychoticism (Bonferroni-adjusted pairwise comparisons for: active NSSI and no NSSI *p*<.001; active NSSI and previous NSSI *p*=.014 (close to significant result)). No significant differences were seen for levels of antagonism, detachment and disinhibition

Figure 2 presents the results of five ANCOVAs conducted for each personality domain and comparing the three groups. Individuals with active NSSI scored higher across all five domains assessed by the PID-5, although only two domains showed significantly higher scores; negative affectivity and psychoticism. Although some departures from normality were observed for the Antagonism model and a modest violation of homogeneity of variance was observed for the Detachment model, no influential observations were identified and the primary conclusions of the study were unaffected.

Negative affectivity differed significantly between the NSSI groups, *F*(2, 224) = 10.33, *p* < .001, η_p_² = 0.08, indicating a medium effect size. Bonferroni-adjusted pairwise comparisons showed that negative affectivity was significantly higher in the active NSSI group than in the no NSSI group (Mdiff = 0.61, SE = 0.14, *p* < .001, 95% CI [0.27, 0.96]), and significantly higher in the active NSSI group than in the previous NSSI group (Mdiff = 0.35, SE = 0.11, *p* = .008, 95% CI [0.07, 0.62]). No significant difference was found between the previous NSSI group and the no NSSI group (*p* = .224).

Psychoticism differed significantly between the NSSI groups (F(2, 224) = 8.91, *p* < .001, ηp² = 0.07), indicating a medium effect size. Bonferroni-adjusted pairwise comparisons showed that psychoticism was significantly higher in the active NSSI group than in the no NSSI group (Mdiff = 0.65, SE = 0.17, *p* < .001, 95% CI [0.25, 1.05]). In addition, psychoticism was close to significantly higher in the active NSSI group than in the previous NSSI group (Mdiff = 0.38, SE = 0.13, *p* = .014, 95% CI [0.06, 0.69]). No significant difference was found between the previous NSSI group and the no NSSI group (*p* = .327).

No significant associations were found between NSSI status and the personality domains of antagonism, detachment, or disinhibition.

The group differences described remained significant after controlling for age, sex assigned at birth, anxiety, and depressive symptoms, indicating that these covariates did not account for the observed associations.

To examine the impact of adjusting for anxiety and depressive symptoms, sensitivity analyses were conducted excluding GAD-7 and MADRS-S from the ANCOVA models. The pattern of results remained largely unchanged. Negative Affectivity remained significantly higher in the active NSSI group compared with both the no NSSI group (Mdiff = 0.93, *p* < .001, 95% CI [0.51, 1.35]) and the previous NSSI group (Mdiff = 0.54, *p* < .001, 95% CI [0.21, 0.88]). Similarly, Psychoticism remained significantly higher in the active NSSI group than in both the no NSSI group (Mdiff = 0.93, *p* < .001, 95% CI [0.49, 1.36]) and the previous NSSI group (Mdiff = 0.54, *p* < .001, 95% CI [0.19, 0.89]). In addition, Detachment became significantly higher in the active NSSI group compared with the no NSSI group (Mdiff = 0.68, *p* < .001, 95% CI [0.24, 1.13]). No significant group differences were observed for Antagonism. Disinhibition showed a difference between the active NSSI and no NSSI groups at the conventional *p* < .05 level (Mdiff = 0.55, *p* = .013), but this did not meet the pre-specified significance threshold of *p* < .01.

## Discussion

The main finding of this study is that individuals with active NSSI scored significantly higher in negative affectivity as compared to those with prior NSSI and no previous history of NSSI and significantly higher in psychoticism as compared to those with no history of NSSI and close to significantly higher than those with a previous history of NSSI. Although the distinction between active and previous NSSI should be interpreted with caution, this approach allowed us to explore whether personality traits differed according to current NSSI status. Significant differences were observed among individuals with active NSSI, whereas no significant differences were detected between those with previous NSSI and those with no history of NSSI.

Our findings were in line with our hypothesis that individuals with active NSSI would exhibit higher scores in negative affectivity [18–20]. Both Negative affectivity and NSSI has been associated with borderline personality disorder (BPD); Negative affectivity as a core trait in the AMPD [14], and NSSI as a core symptom in the categorical model of personality disroders [14] .

In contrast to previous studies suggesting higher levels of Antagonism and Disinhibition among individuals with active NSSI, we found no significant differences in these traits [18, 19, 21]. One possible explanation is the use of a clinical sample that included psychiatric control groups. Previous studies reporting associations between NSSI and these trait domains have primarily been conducted in non-clinical student samples [18, 19, 21]. It is therefore possible that elevated levels of Antagonism and Disinhibition reflect broader psychopathology rather than characteristics specific to NSSI.

At the same time, the AMPD conceptualizes borderline personality disorder as being characterized not only by prominent Negative Affectivity but also by Disinhibition and Antagonism [14]. Against this background, our findings may suggest that active NSSI is more closely associated with negative emotionality than with the broader constellation of maladaptive personality traits typically linked to borderline personality pathology.

Individuals with active NSSI also scored unexpectedly higher in psychoticism in comparison to those with no history of NSSI. This too may reflect the fact that the present sample consisted of psychiatric patients. Within the AMPD framework, psychoticism does not necessarily imply psychotic disorder, but also captures perceptual dysregulation such as depersonalisation, derealization and dissociative experiences [14]. Dissociation has been closely linked to NSSI and together with post traumatic stressdirorder (PTSD) has been proposed to mediate the relationship between trauma and NSSI [45]. Furthermore, in the context of NSSI, these traits may reflect difficulties mentalizing under emotional stress, verbalizing internal experiences, and maintaining a coherent sense of self and others in interpersonal situations [46]. These impairments could contribute to affect dysregulation and increase vulnerability to NSSI as a maladaptive emotion-regulation strategy.

To our knowledge, only one previous study has reported elevated levels of both negative affectivity and psychoticism, conducted in a sample of young adults attending youth mental health services. That study also identified higher levels of detachment and antagonism, findings not replicated in our sample. Although their sample size and age range were comparable to ours, the inclusion of suicidal ideation as a grouping variable may have influenced group composition.

### Strengths and limitations

One strength of this study is its relatively large clinical sample size, using a cohort from two general psychiatric outpatient units, which enhances the generalizability of our findings. Another strength is that we could adjust our findings for confounding variables, including age, sex assigned at birth and levels of depression and anxiety. Both depression and anxiety are facets within the PID-5, with anxiousness being a core component of negative affectivity [32, 33]. Despite this adjustment, our results still demonstrated significantly higher values for negative affectivity and psychoticism in the active NSSI group, indicating the robustness and applicability of our findings.

The most prominent limitation is that we did not include a measure of personality functioning (LPFS) in the data collection. We therefore don’t know if findings reflect a more general severity of psychopathology rather than trait-specific trait associations. Another limitation is that we relied on participants’ self-reported frequency and timing of NSSI, introducing a potential recall bias. Accurate recall of NSSI can be challenging, particularly for individuals with frequent episodes, which may lead to some misclassification and impact the observed relationships across domains [47, 48]. In addition, for the same reasons we chose to extract information about previous suicide attempts from medical records and not from interview with participants. A previous study from an Australian prison reported less than 40% accuracy when comparing self-reported NSSI to information about NSSI extracted from medical records [47]. Optimal would have been to combine reports of NSSI and attempted suicide from sources – self-reported and from medical records.

Furthermore, our sample showed a sex imbalance, which may have influenced the results. Although, results were adjusted for this factor and proved to have no significant effect. Evidence on sex differences in NSSI prevalence is mixed with some suggesting no significant difference [11, 49], some studies suggest higher rates among women [50, 51]. Second, our sample included only participants aged 18–35. Expanding the age range could enhance the generalizability of the findings, however also make it more heterogenous. Nonetheless, it is worth noting that the highest rates of NSSI are reported among individuals aged 14–24 [52]. A further limitation is that the included measures assessed constructs over different timeframes. While the PID-5 captures relatively stable personality traits, symptom measures assessed more recent experiences and the ISAS focused on recent NSSI behaviors. This temporal mismatch may have attenuated some associations and limits conclusions.

Lastly, the cross-sectional design limits our ability to examine changes over time as well as causality. Future longitudinal studies could provide insights into how PID-5 scores evolve with changes in NSSI behavior, such as cessation or relapse.

### Clinical implications and future work

Our findings may have clinical implications, as the PID-5 is already widely used in clinical settings and there is a need to further refine its application in individuals with NSSI. Specifically, the results suggest that elevated Negative Affectivity may be relevant among individuals with ongoing NSSI, irrespective of diagnosis. Assessment of maladaptive personality traits may therefore contribute to a more comprehensive understanding of the emotional vulnerabilities associated with active NSSI.

Longitudinal research is needed to clarify how changes in maladaptive personality traits relate to the course and persistence of NSSI. Future longitudinal studies could also examine the extent to which maladaptive personality traits change over time and whether such changes can be facilitated through treatments grounded in the AMPD framework.

## Conclusion

This study of a clinical sample showed that individuals with active NSSI scored significantly higher on Negative Affectivity than individuals with a previous history of NSSI and those with no history of NSSI. In addition, individuals with active NSSI scored significantly higher on Psychoticism than those with no history of NSSI. These findings contribute to the growing literature on the relationship between self-reported personality traits and NSSI. Future research is needed to determine whether such trait dimensions may help improve our understanding of vulnerability to NSSI and inform prevention and treatment efforts.

## Supplementary Information

Below is the link to the electronic supplementary material.

Supplementary Material 1

Supplementary Material 2

Supplementary Material 3

## Data Availability

The deidentified dataset supporting the conclusions of this article is included within the article and its additional files. The SPSS syntax used to reproduce the reported analyses is also included as an additional file. Data sharing was approved by the Swedish Ethical Review Authority (amendment no. 2026-04635-02), and no data that could enable the identification of an individual participant are included.

## Abbreviations

ADHD: Attention-deficit/hyperactivity disorder
AMPD: Alternative Model for Personality Disorders
BPD: Borderline Personality Disorder
DSM-5: Diagnostic and Statistical Manual of Mental Disorders, Fifth Edition
FFM: Five-factor model
GAD-7: Generalized Anxiety Disorder 7-item scale
ISAS: Inventory of Statements About Self-injury
MADRS-S: Montgomery-Åsberg Depression Rating Scale – Self-administered
MINI 7.0: Mini International Neuropsychiatric Interview 7.0
NSSI: Nonsuicidal Self-Injury
PID-5: Personality Inventory for DSM-5
PTSD: Post-traumatic stress disorder
REDCap: Research Electronic Data Capture
