# Supplementary material for: Elevated Negative Affectivity and Psychoticism in individuals with active Nonsuicidal Self-Injury (NSSI) compared with individuals with previous or no NSSI
Source: Borderline Personal Disord Emot Dysregul. 2026 Sep 22;13:28. doi: 10.1186/s40479-026-00373-z (PMC13599297; doi:10.1186/s40479-026-00373-z)
Supplement: Supplementary file 1 [file 40479_2026_373_MOESM1_ESM.docx]

Supplement

An exploratory multiple linear regression analysis was conducted using log-transformed lifetime NSSI frequency as the dependent variable. The five PID-5 domains, age, and sex were entered simultaneously as predictors. The overall model was significant, F(7,223) = 10.35, p < .001, explaining 24.5% of the variance in lifetime NSSI frequency (R² = .245). Psychoticism showed the strongest positive association with NSSI frequency (β = .204, p = .015), whereas Negative Affectivity showed a trend-level association (β = .135, p = .059). No significant associations were observed for Detachment, Antagonism, or Disinhibition. These findings generally support the primary analyses, although the associations were attenuated when NSSI was modeled as a continuous lifetime frequency variable rather than as a categorical indicator of current NSSI status.

**Supplementary Figure 1. Distribution of lifetime NSSI frequency before and after log transformation.**
Histograms illustrate the distribution of raw NSSI frequency (top) and log-transformed NSSI frequency (ln[NSSI frequency + 1]; bottom). Due to substantial positive skewness in the raw frequency variable, the log-transformed variable was used in the exploratory multiple regression analysis.

**Supplementary Table 1.** Multiple linear regression analysis predicting lifetime NSSI frequency (log-transformed) from PID-5 domains

| **Predictor** | **B** | **SE** | **β** | **95% CI for B** | **p** | **VIF** |
| --- | --- | --- | --- | --- | --- | --- |
| Negative Affectivity | 0.356 | 0.188 | .135 | −0.014, 0.726 | .059 | 1.49 |
| Detachment | 0.285 | 0.171 | .108 | −0.053, 0.622 | .098 | 1.24 |
| Antagonism | 0.149 | 0.175 | .056 | −0.196, 0.495 | .395 | 1.30 |
| Disinhibition | −0.023 | 0.181 | −.009 | −0.381, 0.335 | .899 | 1.39 |
| Psychoticism | 0.540 | 0.220 | .204 | 0.107, 0.974 | .015 | 2.04 |
| Age | 0.015 | 0.035 | .025 | −0.055, 0.085 | .668 | 1.04 |
| Sex | −1.888 | 0.373 | −.310 | −2.623, −1.153 | <.001 | 1.11 |

*Outcome variable was log-transformed lifetime NSSI frequency (ln[NSSI frequency + 1]). β represents standardized regression coefficients. All PID-5 domains were entered simultaneously together with age and sex. VIF = variance inflation factor.*

**Model fit:**
R² = .245, adjusted R² = .221, F(7,223) = 10.35, p < .001.

**Supplementary Figure 2. Association between lifetime NSSI frequency and PID-5 Negative Affectivity.**
Scatterplot showing the association between log-transformed lifetime NSSI frequency (ln[NSSI frequency + 1]) and standardized PID-5 Negative Affectivity scores. The solid line represents the linear regression fit, with shaded areas indicating 95% confidence intervals.

**Supplementary Figure 3. Association between lifetime NSSI frequency and PID-5 Psychoticism.**
Scatterplot showing the association between log-transformed lifetime NSSI frequency (ln[NSSI frequency + 1]) and standardized PID-5 Psychoticism scores. The solid line represents the linear regression fit, with shaded areas indicating 95% confidence intervals.

**Active NSSI**

**Previous NSSI**

**No NSSI**

**Full sample**

**Supplementary Figure 4. Distributions of PID-5 domain scores by NSSI group and in the full sample.** Histograms with overlaid density curves show the distributions of standardized scores for the five PID-5 domains—Negative Affectivity, Detachment, Antagonism, Disinhibition, and Psychoticism—separately for participants with Active NSSI, Previous NSSI, and No NSSI and in the full sample. Higher scores indicate greater expression of maladaptive personality traits.
